# Awake closed manual reduction of cervical spine dislocation as an emergency bridge to surgery: a case report

**DOI:** 10.3389/fmed.2026.1787561

**Published:** 2026-04-16

**Authors:** Fangzheng Lin, Ji Qi, Jing Li, Jianglin Wu, Yu Hou, Minshan Feng, Yongjin Li, Shudong Chen, Dingkun Lin

**Affiliations:** 1 Wangjing Hospital of China Academy of Chinese Medical Sciences, Beijing, China; 2 Key Laboratory of Beijing of TCM Bone Setting, Beijing, China; 3 Orthopaedic Hospital of Guangdong Provincial Hospital of Chinese Medicine, Guangzhou, China; 4 Guangzhou Key Laboratory of Cervical Mechanobiology, Guangzhou, China; 5 Chinese Medicine Guangdong Laboratory, Zhuhai, China; 6 Guangdong Provincial Key Laboratory of Clinical Research on Traditional Chinese Medicine Syndrome, Guangzhou, China

**Keywords:** bridge to surgery, cervical spine dislocation, closed reduction, manipulation, spinal cord injury

## Abstract

**Background:**

Early closed reduction of cervical spine dislocation is essential for spinal cord decompression and neurological preservation. Standard techniques require either general anesthesia for manipulation or specialized traction devices such as Gardner-Wells tongs or halo systems. Awake manual reduction without such equipment or anesthesia has been rarely reported.

**Case presentation:**

A 74-year-old woman sustained a bilateral C5–C6 facet fracture-dislocation with incomplete spinal cord injury (ASIA grade C) following a traffic accident. Approximately 10 h after injury she arrived at our emergency department. Upon arrival, a prompt awake closed reduction was performed using freehand cervical manipulation without traction devices, guided by real-time patient feedback. No analgesia or sedation was administered before or during reduction. The patient remained fully conscious to allow continuous neurological monitoring. Successful reduction was achieved within 15 min with immediate improvement in pain and upper-extremity motor function. The patient subsequently underwent anterior cervical discectomy and fusion and demonstrated satisfactory neurological recovery at the 18-month follow-up.

**Conclusion:**

Awake closed manual reduction may serve as a feasible emergency bridge intervention for cervical fracture-dislocation in time-critical scenarios, such as during preoperative preparation or when the therapeutic window demands immediate decompression. This technique requires experienced practitioners and continuous neurological monitoring, and further studies are warranted to define its safety profile and optimal indications.

## 1 Introduction

Traumatic spinal cord injury is frequently associated with cervical spine dislocation, most commonly following high-energy trauma such as road traffic accidents (1, 2). The subaxial cervical region (C3–C7) is particularly vulnerable and over 50% of cervical spine injuries occur between C5 and C7 (3). Such injuries frequently result in severe neurological deficits. Notably, bilateral facet dislocations at these levels are associated with severe neurological deficits, in a substantial proportion of patients, with over 50% of patients presenting with severe neurological impairment developing tetraplegia (4, 5). Given the high risk of devastating outcomes, early intervention is critical to prevent secondary spinal cord damage and optimize functional recovery.

Early closed reduction (CR) is essential to realign the vertebrae and relieve spinal cord compression as soon as possible (6). Standard CR typically employs incremental skull traction using Gardner-Wells tongs or halo devices. Open reduction is used when CR fails or is contraindicated (7). Despite consensus on the importance of early reduction, several aspects of CR management remain debated. One key controversy concerns whether reduction should be performed with the patient awake or under general anesthesia (GA).

Performing reduction with the patient awake permits continuous neurological examination and immediate detection of deterioration, but it may be limited by pain and inadequate muscle relaxation. In contrast, reduction under GA provides optimal muscle relaxation and patient comfort but precludes real-time clinical feedback, necessitating reliance on intraoperative neuromonitoring (8). Additionally, the technique of CR itself varies considerably. Traction-based protocols using Gardner-Wells tongs or halo devices achieve reduction through progressive axial distraction with equipment and structured weight-loading, whereas device-assisted manipulative techniques combine traction with controlled rotational or translational maneuvers performed by experienced clinicians (7, 9, 10). However, most reported awake reduction techniques still rely on skeletal traction devices such as Gardner-Wells tongs or halo systems. Truly device-free manual reduction performed without cranial traction devices or general anesthesia has rarely been described in the literature.

In this context, we report a case of subaxial cervical spine dislocation in a 74-year-old woman who presented with severe neurological impairment. She underwent awake CR by freehand manipulation without cranial traction devices as an emergency bridge during preoperative preparation, followed by anterior cervical discectomy and fusion at C5/C6. This case report does not propose manual reduction as a replacement for established traction-based or surgical reduction techniques; rather, it illustrates how a carefully executed manual reduction, performed by an experienced spine surgeon in a strictly monitored setting, may serve as a feasible adjunctive option when conventional traction is not immediately available. We also review the current literature on the management of subaxial cervical fracture–dislocations, with a particular focus on the role, opportunities, and limitations of CR strategies.

## 2 Case presentation

**Figure 1:**
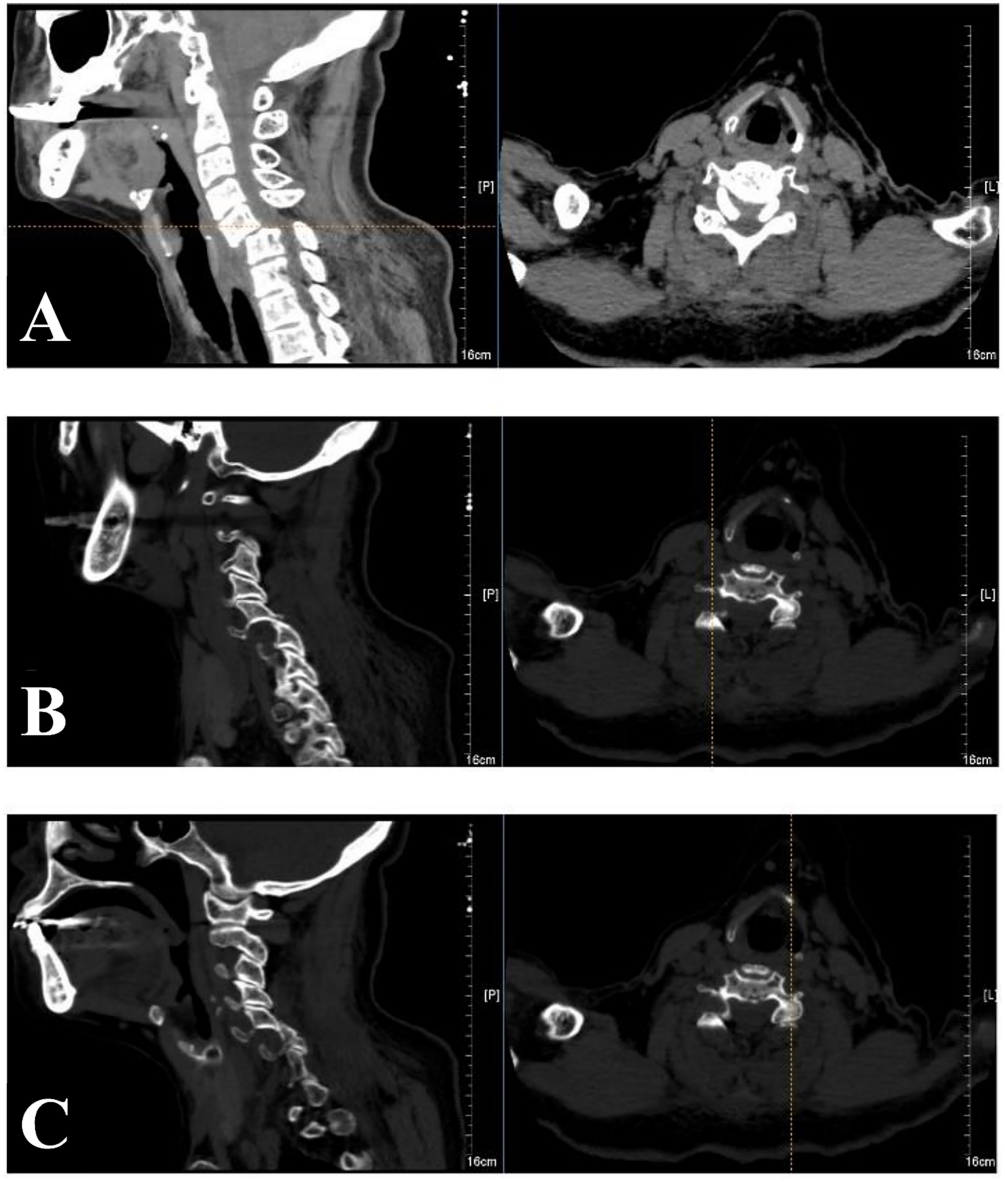
Sagittal CT images of the cervical spine demonstrating bilateral C5–C6 facet dislocation with anterior translation of C5. **(A)** Midsagittal images. **(B)** CT image of the right articular process. **(C)** CT image of the left articular process.

A 74-year-old female patient sustained a cervical spine injury in a road traffic accident. Initial cervical computed tomography (CT) was performed after the trauma (Figure 1). CT demonstrated a bilateral facet dislocation at the C5–C6 level, consistent with a distractive flexion injury mechanism. Approximately 10 h post-injury, she was transferred to our emergency department for further management. She was transported by ambulance with cervical spine immobilization using a rigid collar and spine board. No reduction attempts or traction were performed at the former hospital. On arrival, pre-reduction magnetic resonance imaging (MRI) was performed, which confirmed bilateral C5–C6 facet dislocation with significant spinal canal stenosis secondary to locked facet joints. No large disc herniation or epidural hematoma was detected. Intramedullary high-signal intensity T2-weighted imaging was consistent with spinal cord contusion, with no evidence of cord transection. This examination was conducted to assess disc herniation and spinal cord compression, characterize injury morphology, and rule out contraindications to CR.

She complained of severe neck pain (visual analog scale, VAS, 6) with radiating upper-extremity pain (VAS, 5), accompanied by numbness in all four limbs. Neurological examination revealed preserved sensation above the xiphoid process and over the palmar and radial regions of both hands, with absent sensation elsewhere. Motor testing revealed marked weakness in the upper extremities (Medical Research Council grade 2/5) and complete paralysis of both lower extremities (grade 0/5). Deep tendon reflexes were absent bilaterally. Overall, the findings were consistent with an incomplete cervical spinal cord injury, classified as American Spinal Injury Association (ASIA) Impairment Scale grade C and Frankel grade C (Table 1).

**Table 1:** Comparison of VAS scores, muscle strength, and ASIA grade pre- and post-treatment.

Clinical indicators		Physical examination		Time points of clinical evaluation		
			Pre-CR	Post-CR	Post-operation	18 months after surgery
VAS scores		Neck	6	2	1	0
		Radicular pain of the upper limbs	5	2	1	0
Muscle strength	Deltoid	Right	2	3	Same as post-reduction	4
		Left	2	2		4
	Bicipital	Right	2	3		4
		Left	1	2		4
	Triceps brachii	Right	1	2		3
		Left	1	2		3
	Flexor muscles of the wrist	Right	0	0		2
		Left	0	0		2
	Lower limbs		0	0		0
AISA grade			C	C	C	C

Considering the prolonged interval since injury (approximately 10 h) and the risk of ongoing spinal cord compromise from sustained compression, CR was performed expeditiously upon completion of the pre-reduction evaluation to minimize further secondary neurological injury. The patient remained fully conscious throughout the procedure, enabling continuous neurological monitoring via real-time verbal communication to promptly detect any deterioration. The reduction was performed by an experienced spine surgeon with extensive expertise in spinal manipulation, with patient positioned supine with her head and neck slightly overhanging the edge of the bed. No dedicated assistant was assigned to stabilize the patient’s shoulders or torso, as the patient’s body weight and mattress friction provided sufficient passive counter-traction. Traction force was not quantitatively measured but was continuously titrated throughout the procedure according to three real-time signals: the patient’s verbal pain reports, the surgeon’s tactile perception of muscular resistance, and ongoing assessment of neurological status. The surgeon applied bimanual control at the occiput and mandible, enabling sequential flexion traction followed by an extension-assisted reduction. Additional medical staff were present to assist with positioning, monitor vital signs, and document neurological status. The surgical team was on standby for emergent open reduction if needed. No analgesia or sedation was administered, and the patient remained fully awake and verbally communicative throughout.

**Figure 2:**
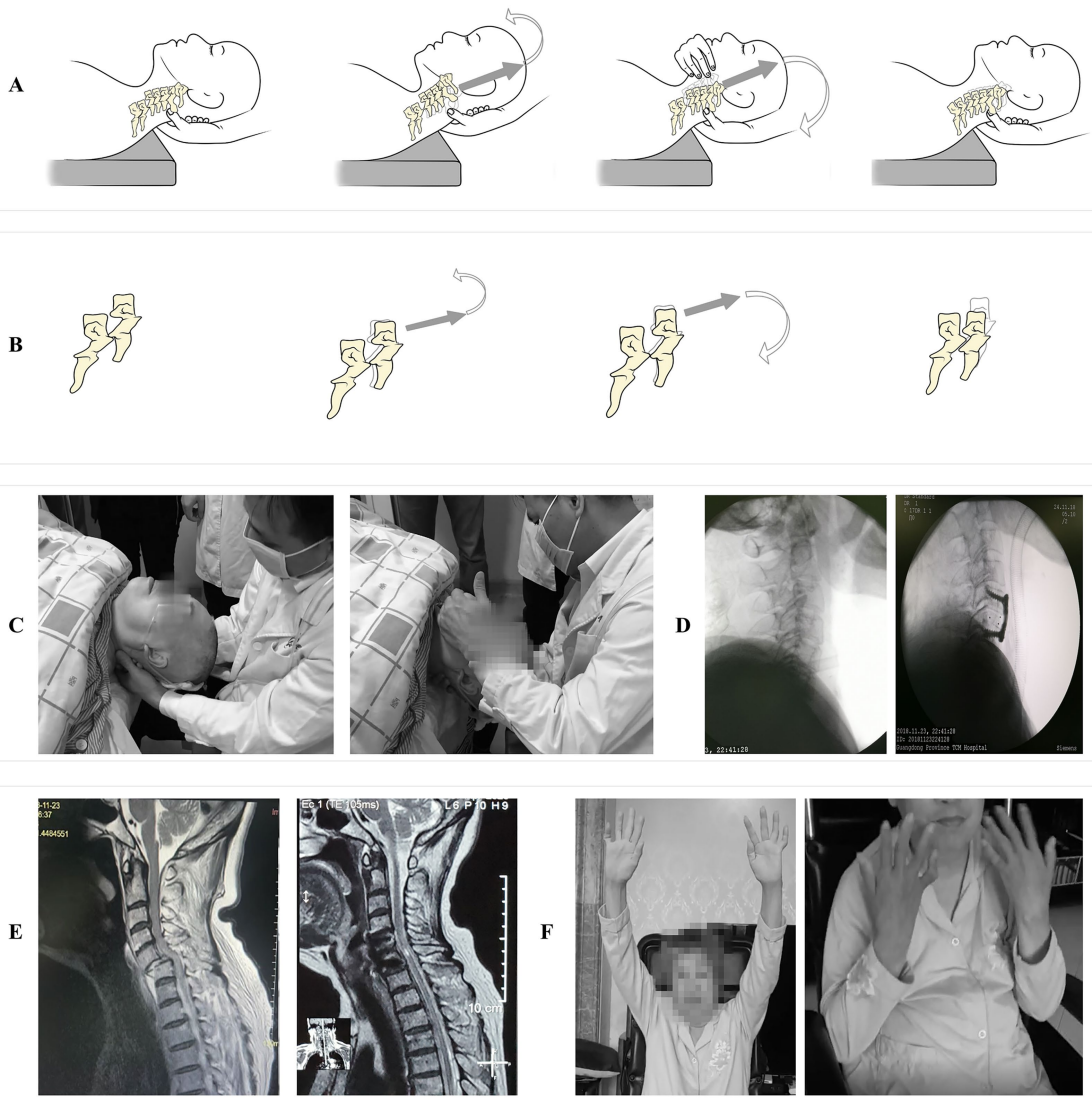
Procedural steps of manual closed reduction for cervical spine dislocation and 18-month follow-up image. **(A)** Schematic diagram of manual closed reduction technique: from dislocation position, through flexion (unlocking) to extension (reduction), then restore cervical spine alignment. **(B)** Schematic diagram of detailed sequence of closed reduction for C5–C6 dislocation. **(C)** Clinical application of the awake manual reduction technique. **(D)** Post-reduction and postoperative C-arm image. **(E)** Pre-reduction sagittal T2-weighted MRI showing C5–C6 dislocation and spinal cord compression, 18-month follow-up MRI image showing complete spinal cord decompression and restoration of spinal alignment. **(F)** Significant recovery of bilateral upper limb motor function at 18-month follow-up.

Given the distractive flexion injury pattern, the cervical spine was initially maintained in flexion to facilitate facet unlocking while minimizing the risk of secondary spinal cord injury. Under sustained axial traction along the axis of deformity, minimal controlled rotational adjustments were attempted to determine whether the locked facets had reached a tip-to-tip apposition. The reduction was then completed by gradual transition into extension to restore C5–C6 segmental alignment. An audible “click” was noted, and palpation confirmed restoration of normal spinous process alignment, suggesting successful realignment of the C5 vertebra. C-arm fluoroscopy was immediately employed to confirm successful realignment of the C5–C6 facet joints and restoration of normal cervical spine alignment prior to any patient repositioning or induction of general anesthesia for definitive surgical stabilization (Figure 2). The entire CR procedure was completed within 15 min. Immediately post-reduction, the patient reported marked pain relief: neck pain decreased from VAS 6 to 2 and radicular pain from VAS 5 to 2 (Table 1). Upper-extremity motor strength improved concurrently. C-arm fluoroscopy subsequently confirmed successful reduction. The patient’s vital signs were stable and normal throughout the procedure, with no neurological deficits detected. The patient subsequently underwent anterior cervical discectomy and fusion at C5/6 under GA. Postoperatively, her neurological function continued to improve progressively, with no new deficits observed. At the 18-month follow-up the patient reported complete resolution of neck and radicular pain, with upper-limb strength improved to grade 4/5 in the deltoid and biceps bilaterally, grade 3/5 in the triceps, and grade 2/5 in the wrist flexors. Radiographic imaging confirmed solid intervertebral fusion and an intact instrumentation construct (Figure 2).

## 3 Discussion

Early realignment and stabilization for cervical spine dislocation is critical to prevent secondary spinal cord damage and optimize neurological recovery (6, 11–13). Current evidence supports achieving reduction/decompression as early as feasible, with an established ideal window of within 24 h of injury (and even greater neurological benefits reported with ultra-early intervention within 6 h), particularly for patients with incomplete spinal cord injury (11, 14–16). In practice, however, timing and approach to reduction can vary widely due to systemic and regional factors. For example, delays in acute management have been observed in some regions, including China, due to resource or referral challenges (17). At the same time, patient-specific factors (age, comorbidities, injury morphology, etc.) influence recovery, underscoring the need for individualized treatment plans (18). In our case, an immediate manual CR was performed without anesthesia upon emergency admission (approximately 10 h post-injury, during surgical preparation). This method decompressed the cord prior to surgical treatment, and the follow-up results verified that this emergency CR may serve as a practical reference for individuals with analogous clinical conditions.

Whether to perform CR with the patient awake or under GA remains controversial (19). Awake CR allows continuous neurological monitoring through real-time patient feedback, permitting immediate detection of neurological deterioration and prompt cessation of the maneuver if necessary (7, 8). Multiple studies have demonstrated the safety and efficacy of this approach, with reported success rates of 90–98% and neurological deterioration rates as low as 1.3% (20–23) (Table 2). Notably, most reported awake CR techniques use skeletal traction devices such as Gardner-Wells tongs or halo crowns, which require specialized equipment and incremental weight application. These findings collectively support the safety of this approach, with the key advantage being continuous neurological monitoring throughout the procedure. By contrast, performing CR under GA provides complete muscle relaxation and lack of patient cooperation, which may facilitate the reduction in cases of severe pain, muscle spasm, or patient uncooperativeness (7, 24). However, GA eliminates protective patient feedback and introduces procedural risks. For example, intubation often requires near-full cervical extension, which in patients with unreduced cervical spine dislocations (especially flexion-compression injuries) can exacerbate cord compression (25). Although neurological deterioration directly attributable to intubation remains rare and difficult to prove causally, case reports have documented catastrophic outcomes including new-onset quadriplegia following emergent intubation in patients with unstable cervical spines (26, 27). In the present case, given the patient’s alertness and cooperation, we proceeded with awake manual CR. Her real-time feedback effectively served as continuous neurological monitoring, allowing us to modulate our maneuvers accordingly and cease immediately if any new symptoms emerged. Ultimately, the choice between awake and anesthetized approaches should be individualized based on patient consciousness, cooperation, pain tolerance, injury pattern, time since injury, team expertise, and available monitoring resources (7, 21).

**Table 2:** Summary of studies reporting closed reduction times for cervical spine dislocations.

References	Sample size	Closed reduction method	Technique	Anesthesia	Success rate	Neurological deterioration
Lee et al. (28)	119 (traction)/91 (MUA)	Cranial traction/MUA	Group A: Skull traction Group B: MUA	Awake/GA	Group A: 88% Group B: 73%	Lower in Group A
Xiong et al. (24)	31	MUA	Manipulation without device	GA	74%	0%
Grant et al. (20)	82	Cranial traction	Gardner-Wells tong	Awake (sedation)	97.6%	NR
Miao et al. (29)	40	Cranial traction	Skull traction	Awake	95%	0%
Sousa et al. (22)	59	Cranial traction with manual-assisted	Gardner-Wells tongs	Awake	88%	NR
Oae et al. (21)	40	Cranial traction	Halo crown + motorized bed	Awake	90%	0% (transient in 3, reversed)

MUA, manipulation under anesthesia; GA, general anesthesia; NR, not reported.

Various CR techniques for cervical spine dislocation rely on cranial traction devices such as Gardner-Wells tongs or halo crown, with reduction times ranging from 21 min to over 2 h (20, 22, 24, 28, 29). Although traction-based CR is widely used, it requires specialized equipment and careful incremental weight application. Complications such as pin loosening (19–36%), pin-site infection (6.3–20%), severe pin discomfort (18%), neck pain (20.7%), and dysphagia (1.7%) have been reported, and prolonged reduction time may delay definitive decompression (30–32). In the present case, the patient arrived approximately 10 h post-injury with sustained spinal cord compression, necessitating expeditious intervention. The freehand manual reduction was achieved within 15 min without specialized equipment or GA, thereby avoiding equipment-related delays and potential complications from skeletal traction. The fundamental biomechanical principles of successful CR remain consistent with or without traction devices. The core maneuvers follow a sequential process: traction along the axis of deformity in flexion to unlock the facets, gentle rotation to disengage the articular processes, and gradual extension to restore alignment. A “click” that is typically heard or felt may indicate successful CR (19). For bilateral dislocations, Perez Rios et al. (32) specified that while maintaining traction with flexion, the head is rotated 30° to 45° to one side, returned to midline, then rotated 30° to 45° to the opposite side before proceeding to extension. In our case of bilateral C5–C6 facet dislocation, we employed the same flexion–rotation–extension sequence without traction devices. The critical difference from device-assisted techniques lies in the demands of the surgeon: without mechanical traction to provide sustained distraction, the clinician must maintain precise manual control over reduction angles, hand stability and timely minor adjustment throughout the maneuver. Our team’s prior experience with cervical angled manual traction (33) provided essential technical familiarity for this emergency application.

A critical risk of any closed reduction is posterior displacement of disc fragments causing acute cord compression; pre-reduction MRI to exclude significant disc herniation is therefore mandatory before any manipulation is attempted, and when large herniation is present, upfront anterior decompression should be considered as a safer alternative. Unlike traction-based methods that apply incremental quantifiable loads, freehand reduction relies entirely on the surgeon’s tactile judgment to estimate force magnitude and direction, making standardization impossible and operator expertise the primary determinant of safety.

Given the patient’s incomplete injury after a prolonged pre-hospital delay and ongoing cord compromise risk, we elected to perform awake manual reduction as an emergency bridge to surgery. The decision was based on urgent circumstances rather than routine preference. Although upfront anterior decompression represents a viable alternative when large disc herniation is present on MRI, no such herniation was identified in our patient, and the immediate bedside nature of CR offered a critical time advantage over surgical preparation in a case of ongoing cord compression (6, 25); the surgical team remained on standby for immediate open reduction throughout. This report does not propose manual reduction as a replacement for established traction-based or surgical reduction techniques. Instead, it documents one instance in which, driven by the urgent clinical need to decompress the spinal cord when conventional traction was not immediately available, awake manual reduction performed by an experienced surgeon with surgical backup appeared to achieve satisfactory bridging reduction. Surgeons contemplating this maneuver must recognize that posterior displacement of disc fragments during reduction can cause acute cord compression and sudden neurological deterioration; pre-reduction MRI to exclude significant disc herniation is therefore mandatory, and when large herniation is identified, upfront anterior decompression represents a safer alternative. Additional prerequisites include feasibility of continuous neurological monitoring via verbal communication, absence of posterior element fractures, and immediate surgical backup for conversion to open reduction if needed.

Freehand CR without any traction devices or GA remains uncommonly reported, and our case adds to the limited evidence supporting its feasibility in selected circumstances. It should be acknowledged that neurological recovery following traumatic cervical spine injury is influenced by multiple factors beyond the reduction technique itself. From a prognostic perspective, factors associated with better neurological recovery include incomplete injury (ASIA grade C or D), central cord syndrome, higher cervical injury level, and early surgical decompression (≤24 h), while older age, delayed surgery, and comorbidities are negative predictors (16, 18, 34). Therefore, for elderly patients with delayed presentation, a rapid bedside reduction strategy may be particularly valuable to minimize ongoing cord compression while preparing for definitive stabilization.

This case carries several clinical implications. First, bedside manual CR may serve as a bridge to definitive surgery, allowing patients to enter the operating room in a more favorable neurological state—aligning with the paradigm shift toward early intervention (11, 14). Second, this approach may offer a time-saving alternative when conventional traction devices are unavailable or when transfer delays preclude timely decompression. To prevent inappropriate generalization, we propose the following framework for patient selection. Circumstances in which awake freehand manual CR may be considered include: (1) incomplete spinal cord injury; (2) absence of significant disc herniation or epidural hematoma on pre-reduction MRI; (3) an awake, alert, and cooperative patient capable of providing continuous real-time neurological feedback; and (4) availability of an experienced spine surgeon with specific expertise in cervical manipulation, supported by a team prepared for immediate surgical conversion. Circumstances requiring caution or representing relative contraindications include: (1) complete spinal cord injury (ASIA grade A or B); (2) significant posterior bony fragments or comminuted fracture patterns that may be displaced during manipulation; (3) severe multi-level instability; (4) inability to maintain continuous neurological monitoring due to altered consciousness or uncooperativeness; and (5) absence of immediate surgical backup for conversion to open reduction if needed. Limitations of this report include its single-case nature and absence of comparative data against traction-based or anesthesia-assisted reduction. Larger prospective studies are required to establish standardized protocols and define optimal indications.”

## 4 Conclusion

This case demonstrates that awake manual closed reduction, performed approximately 10 h post-injury, can achieve prompt spinal cord decompression without traction devices or anesthesia, offering a valuable alternative in time-critical scenarios such as conventional traction devices are unavailable, transfer delays or while awaiting definitive surgery. This technique requires substantial spine surgeon expertise and continuous neurological monitoring and should not be considered a routine replacement for skeletal traction or surgical reduction. Prospective studies are warranted to establish standardized protocols and define optimal indications.

## Data Availability

The original contributions presented in the study are included in the article/supplementary material, further inquiries can be directed to the corresponding authors.
